# Aqua­azido­{3,3′-[*o*-phenyl­enebis(nitrilo­methyl­idyne)]di-2-naphtholato}manganese(III)

**DOI:** 10.1107/S1600536809052659

**Published:** 2009-12-12

**Authors:** Cui-Juan Wang, Xian-Li Zhou, Bing-Jun Zhang

**Affiliations:** a Department of Chemistry and Chemical Engineering, School of Bioengineering, SouthWest JiaoTong University, Chengdu, Sichuan 610031, People’s Republic of China

## Abstract

In the title complex, [Mn(C_28_H_18_N_2_O_2_)(N_3_)(H_2_O)], the Mn^III^ ion adopts a distorted *fac*-MnO_3_N_3_ octa­hedral geometry arising from the *O*,*N*,*N*′,*O*′-tetra­dentate Schiff base ligand, an azide ion and a water mol­ecule. In the crystal, inter­molecular O—H⋯(O,O) and O—H⋯N hydrogen bonds and π–π inter­actions [centroid–centroid separation = 3.5535 (13) Å] link the mol­ecules into chains.

## Related literature

For background to Schiff base–metal complexes, see: Sunatsuki *et al.* (2002 ▶).
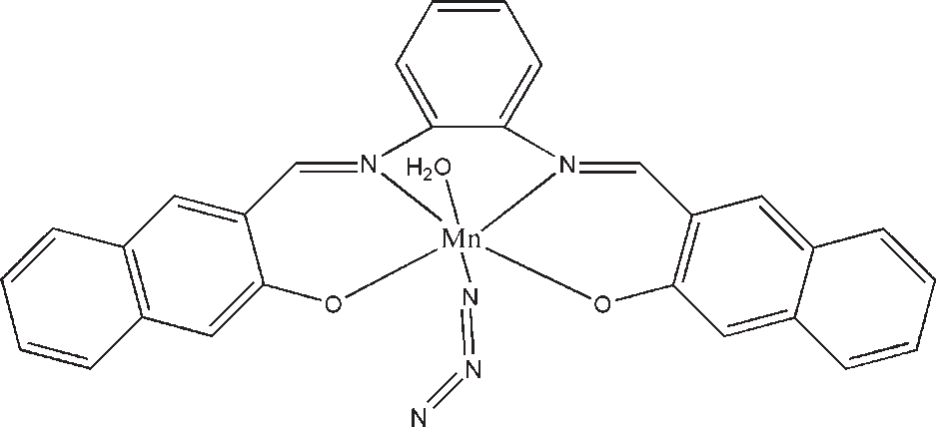


## Experimental

### 

#### Crystal data

[Mn(C_28_H_18_N_2_O_2_)(N_3_)(H_2_O)]
*M* *_r_* = 529.43
Triclinic, 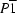

*a* = 6.6827 (1) Å
*b* = 11.8803 (2) Å
*c* = 15.3778 (3) Å
α = 99.455 (1)°
β = 97.692 (1)°
γ = 98.180 (1)°
*V* = 1176.49 (4) Å^3^
*Z* = 2
Mo *K*α radiation
μ = 0.60 mm^−1^
*T* = 298 K
0.29 × 0.20 × 0.12 mm

#### Data collection

Bruker APEXII CCD diffractometer
Absorption correction: multi-scan (*SADABS*; Bruker, 2005 ▶) *T*_min_ = 0.844, *T*_max_ = 0.931
12984 measured reflections
4205 independent reflections
3519 reflections with *I* > 2σ(*I*)
*R*_int_ = 0.025

#### Refinement

*R*[*F* ^2^ > 2σ(*F* ^2^)] = 0.035
*wR*(*F* ^2^) = 0.121
*S* = 0.88
4205 reflections
340 parameters
3 restraints
H atoms treated by a mixture of independent and constrained refinement
Δρ_max_ = 0.26 e Å^−3^
Δρ_min_ = −0.23 e Å^−3^

### 

Data collection: *APEX2* (Bruker, 2005 ▶); cell refinement: *SAINT* (Bruker, 2005 ▶); data reduction: *SAINT*; program(s) used to solve structure: *SHELXS97* (Sheldrick, 2008 ▶); program(s) used to refine structure: *SHELXL97* (Sheldrick, 2008 ▶); molecular graphics: *ORTEP-3* (Farrugia, 1997 ▶) and *PLATON* (Spek, 2009 ▶); software used to prepare material for publication: *SHELXL97*.

## Supplementary Material

Crystal structure: contains datablocks I, global. DOI: 10.1107/S1600536809052659/hb5265sup1.cif

Structure factors: contains datablocks I. DOI: 10.1107/S1600536809052659/hb5265Isup2.hkl

Additional supplementary materials:  crystallographic information; 3D view; checkCIF report

## Figures and Tables

**Table 1:** Selected bond lengths (Å)

Mn1—O2	1.8644 (15)
Mn1—O1	1.8799 (14)
Mn1—N1	1.9663 (17)
Mn1—N2	1.9672 (17)
Mn1—N3	2.2465 (19)
Mn1—O3	2.3905 (16)

**Table 2:** Hydrogen-bond geometry (Å, °)

*D*—H⋯*A*	*D*—H	H⋯*A*	*D*⋯*A*	*D*—H⋯*A*
O3—H3*WA*⋯N3^i^	0.82 (1)	2.10 (3)	2.911 (3)	167 (4)
O3—H3*WB*⋯O1^ii^	0.82 (3)	2.14 (3)	2.941 (2)	164 (4)
O3—H3*WB*⋯O2^ii^	0.82 (3)	2.57 (3)	3.130 (2)	127 (3)

Symmetry codes: (i) 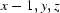; (ii) 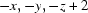.

**Fig. 1.**
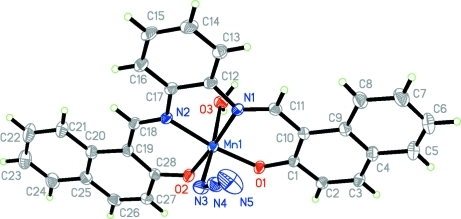
Molecular view of (I) with ellipsoids drawn at the the 30% probability level. H atoms are shown as spheres of arbitrary radius.

## supplementary crystallographic information

### Comment

In recent years, there has been considerable interest in the chemistry of transition metal complexes of Schiff bases (Sunatsuki *et al.*,  2002). In this paper, we report here the synthesis and crystal structure of the title complex (I).

The molecular structure of (I) is illustrated in Fig. 1. The Mn^III^ ion takes a slightly distorted octahedral geometry, where the equatorial plane comprise the two N atoms two imine nitrogen atoms and two O atoms of alkoxide groups. The apical postiions are occupied by the N atom of azide ligand and one O atom from water molecule. The Mn—N(azide) bond length is significantly longer than the Mn-N(imine) (Table 1) and the bond angles deviate from the ideal values (the largest angle is 173.80 (7)°). The chelate bite angles in the five-membered and the six-membered rings formed by the coordination of alkoxide-O and imine-N, and two related imine-N of Schiff base to the Mn^III^ center lie in same plane and are almost parallel to the naphthalen rings with 2.6°.

The structure is further stabilized by strongly π-π stacking interactions between two adjacent naphthalen rings in an offset arrangement. The distance between the centroids of the six-membered rings is 3.55 (3)Å. In addition, Intermolecular O-H···O, O-H···N hydrogen bonds form a zig-zag like chain parallel to the b axis (Table 2).

### Experimental

A mixture of Mn(Ac)_3_, NaN_3_ and 3-((1E)-((E)-2-((naphthalen-2-yl)methyleneamino) phenylimino)methyl)naphthalen-2,2'-diol in water (30mL) was refluxed for 5 hours and then filtered while hot. Colourless blocks of (I) were obtained by evaporating the filtrate at room temperature for a period of three weeks. The compound is insoluble in commom organic solvents and dissolves water very slowly.

### Refinement

The H atoms of organic ligand were placed in calculated positions (C-H = 0.93Å) refined using a riding model, with U_iso_(H) = 1.2U_eq_(C) H atoms of water molecules were located in a difference map and refined with restraints of O-H=0.83 (1)Å, and with U_iso_(H) = 1.5U_eq_(O).

### Crystal data

**Table:**

[Mn(C_28_H_18_N_2_O_2_)(N_3_)(H_2_O)]	*Z* = 2
*M**_r_* = 529.43	*F*(000) = 544
Triclinic, *P*1	*D*_x_ = 1.495 Mg m^−^^3^
Hall symbol: -P 1	Mo *K*α radiation, λ = 0.71073 Å
*a* = 6.6827 (1) Å	Cell parameters from 4205 reflections
*b* = 11.8803 (2) Å	θ = 2.2–25.2°
*c* = 15.3778 (3) Å	µ = 0.60 mm^−^^1^
α = 99.455 (1)°	*T* = 298 K
β = 97.692 (1)°	Block, colourless
γ = 98.180 (1)°	0.29 × 0.20 × 0.12 mm
*V* = 1176.49 (4) Å^3^	

### Data collection

**Table:**

Bruker APEXII CCD diffractometer	4205 independent reflections
Radiation source: fine-focus sealed tube	3519 reflections with *I* > 2σ(*I*)
graphite	*R*_int_ = 0.025
φ and ω scan	θ_max_ = 25.2°, θ_min_ = 2.0°
Absorption correction: multi-scan (*SADABS*; Bruker, 2005)	*h* = −8→8
*T*_min_ = 0.844, *T*_max_ = 0.931	*k* = −14→13
12984 measured reflections	*l* = −18→18

### Refinement

**Table:**

Refinement on *F*^2^	Primary atom site location: structure-invariant direct methods
Least-squares matrix: full	Secondary atom site location: difference Fourier map
*R*[*F*^2^ > 2σ(*F*^2^)] = 0.035	Hydrogen site location: inferred from neighbouring sites
*wR*(*F*^2^) = 0.121	H atoms treated by a mixture of independent and constrained refinement
*S* = 0.88	*w* = 1/[σ^2^(*F*_o_^2^) + (0.1*P*)^2^ + 0.1339*P*] where *P* = (*F*_o_^2^ + 2*F*_c_^2^)/3
4205 reflections	(Δ/σ)_max_ = 0.001
340 parameters	Δρ_max_ = 0.26 e Å^−^^3^
3 restraints	Δρ_min_ = −0.23 e Å^−^^3^

### Special details

**Table:**

Geometry. All esds (except the esd in the dihedral angle between two l.s. planes) are estimated using the full covariance matrix. The cell esds are taken into account individually in the estimation of esds in distances, angles and torsion angles; correlations between esds in cell parameters are only used when they are defined by crystal symmetry. An approximate (isotropic) treatment of cell esds is used for estimating esds involving l.s. planes.
Refinement. Refinement of F^2^ against ALL reflections. The weighted R-factor wR and goodness of fit S are based on F^2^, conventional R-factors R are based on F, with F set to zero for negative F^2^. The threshold expression of F^2^ > 2sigma(F^2^) is used only for calculating R-factors(gt) etc. and is not relevant to the choice of reflections for refinement. R-factors based on F^2^ are statistically about twice as large as those based on F, and R- factors based on ALL data will be even larger.

### Fractional atomic coordinates and isotropic or equivalent isotropic displacement parameters (Å^2^)

**Table:**

	*x*	*y*	*z*	*U*_iso_*/*U*_eq_	
Mn1	0.21230 (5)	0.16035 (2)	0.939323 (19)	0.03163 (14)	
N2	0.2571 (3)	0.32332 (14)	0.99968 (12)	0.0311 (4)	
O1	0.1336 (2)	0.00645 (12)	0.87811 (10)	0.0356 (4)	
N1	0.0823 (3)	0.22059 (14)	0.83821 (12)	0.0307 (4)	
O2	0.3187 (2)	0.11226 (12)	1.04281 (10)	0.0378 (4)	
C11	0.0019 (3)	0.15759 (19)	0.76023 (15)	0.0337 (5)	
H11	−0.0463	0.1971	0.7165	0.040*	
O3	−0.1107 (2)	0.14771 (13)	0.99088 (12)	0.0411 (4)	
C1	0.0450 (3)	−0.03427 (17)	0.79550 (14)	0.0303 (5)	
C28	0.3879 (3)	0.17238 (18)	1.12325 (14)	0.0322 (5)	
C19	0.3971 (3)	0.29292 (19)	1.14745 (14)	0.0339 (5)	
C10	−0.0201 (3)	0.03561 (18)	0.73472 (14)	0.0317 (5)	
C16	0.2261 (4)	0.51867 (18)	0.96493 (16)	0.0396 (5)	
H16	0.2947	0.5559	1.0212	0.048*	
C18	0.3317 (3)	0.36058 (18)	1.08433 (15)	0.0335 (5)	
H18	0.3430	0.4396	1.1054	0.040*	
N3	0.5156 (3)	0.19321 (18)	0.89218 (13)	0.0432 (5)	
C22	0.5792 (5)	0.5194 (3)	1.35685 (19)	0.0701 (9)	
H22	0.5873	0.5986	1.3757	0.084*	
C25	0.5511 (4)	0.2830 (2)	1.30092 (16)	0.0426 (6)	
C12	0.0973 (3)	0.34341 (17)	0.85631 (15)	0.0340 (5)	
C9	−0.1130 (3)	−0.0194 (2)	0.64364 (15)	0.0369 (5)	
C4	−0.1307 (3)	−0.1403 (2)	0.61810 (15)	0.0401 (5)	
C5	−0.2167 (4)	−0.1956 (2)	0.52954 (17)	0.0526 (7)	
H5	−0.2277	−0.2754	0.5134	0.063*	
C3	−0.0665 (3)	−0.20654 (19)	0.68215 (15)	0.0391 (5)	
H3	−0.0817	−0.2865	0.6652	0.047*	
C2	0.0163 (3)	−0.15628 (18)	0.76743 (14)	0.0341 (5)	
H2	0.0549	−0.2023	0.8081	0.041*	
C21	0.4955 (4)	0.4698 (2)	1.27051 (18)	0.0534 (7)	
H21	0.4480	0.5161	1.2318	0.064*	
C20	0.4797 (3)	0.3504 (2)	1.23912 (15)	0.0388 (5)	
C6	−0.2835 (4)	−0.1338 (3)	0.46767 (18)	0.0615 (8)	
H6	−0.3387	−0.1706	0.4095	0.074*	
C14	0.0525 (4)	0.5279 (2)	0.82062 (18)	0.0484 (6)	
H14	0.0026	0.5718	0.7804	0.058*	
C15	0.1542 (4)	0.58246 (19)	0.90428 (17)	0.0435 (6)	
H15	0.1742	0.6628	0.9197	0.052*	
C26	0.5337 (4)	0.1611 (2)	1.27266 (16)	0.0429 (6)	
H26	0.5778	0.1170	1.3138	0.051*	
C13	0.0239 (4)	0.4089 (2)	0.79603 (17)	0.0453 (6)	
H13	−0.0441	0.3728	0.7394	0.054*	
C17	0.1963 (3)	0.39877 (17)	0.94229 (14)	0.0323 (5)	
C8	−0.1867 (4)	0.0416 (2)	0.57819 (17)	0.0507 (6)	
H8	−0.1800	0.1213	0.5932	0.061*	
C27	0.4558 (3)	0.1080 (2)	1.18858 (15)	0.0383 (5)	
H27	0.4460	0.0282	1.1727	0.046*	
C24	0.6383 (4)	0.3370 (3)	1.38941 (17)	0.0570 (7)	
H24	0.6868	0.2925	1.4294	0.068*	
C7	−0.2677 (4)	−0.0144 (3)	0.49312 (17)	0.0589 (7)	
H7	−0.3134	0.0283	0.4512	0.071*	
C23	0.6525 (5)	0.4530 (3)	1.41707 (19)	0.0712 (9)	
H23	0.7105	0.4877	1.4754	0.085*	
N4	0.5327 (3)	0.21532 (17)	0.82123 (15)	0.0454 (5)	
N5	0.5512 (4)	0.2366 (3)	0.75124 (19)	0.0928 (10)	
H3WA	−0.218 (3)	0.149 (3)	0.958 (2)	0.139*	
H3WB	−0.129 (6)	0.095 (3)	1.019 (2)	0.139*	

### Atomic displacement parameters (Å^2^)

**Table:**

	*U*^11^	*U*^22^	*U*^33^	*U*^12^	*U*^13^	*U*^23^
Mn1	0.0397 (2)	0.0212 (2)	0.0312 (2)	0.00640 (14)	−0.00187 (14)	0.00203 (13)
N2	0.0304 (9)	0.0229 (8)	0.0391 (10)	0.0044 (7)	0.0049 (8)	0.0039 (7)
O1	0.0485 (9)	0.0231 (7)	0.0323 (8)	0.0081 (6)	−0.0029 (7)	0.0024 (6)
N1	0.0336 (9)	0.0223 (9)	0.0356 (10)	0.0045 (7)	0.0035 (8)	0.0052 (7)
O2	0.0491 (9)	0.0282 (8)	0.0333 (8)	0.0083 (7)	−0.0016 (7)	0.0030 (6)
C11	0.0342 (11)	0.0340 (12)	0.0343 (12)	0.0075 (9)	0.0026 (9)	0.0108 (9)
O3	0.0420 (9)	0.0340 (9)	0.0492 (10)	0.0069 (7)	0.0057 (7)	0.0137 (7)
C1	0.0284 (10)	0.0281 (10)	0.0324 (11)	0.0036 (8)	0.0045 (9)	0.0014 (9)
C28	0.0279 (10)	0.0342 (12)	0.0331 (11)	0.0046 (8)	0.0046 (9)	0.0034 (9)
C19	0.0314 (11)	0.0334 (12)	0.0343 (12)	0.0018 (9)	0.0054 (9)	0.0022 (9)
C10	0.0313 (11)	0.0308 (11)	0.0311 (11)	0.0040 (8)	0.0024 (9)	0.0033 (9)
C16	0.0421 (12)	0.0278 (11)	0.0495 (14)	0.0055 (9)	0.0125 (11)	0.0049 (10)
C18	0.0308 (11)	0.0266 (10)	0.0399 (12)	0.0016 (8)	0.0075 (9)	−0.0015 (9)
N3	0.0398 (11)	0.0522 (12)	0.0410 (12)	0.0148 (9)	0.0072 (9)	0.0120 (9)
C22	0.092 (2)	0.0518 (17)	0.0509 (17)	0.0014 (16)	0.0000 (16)	−0.0164 (14)
C25	0.0356 (12)	0.0539 (15)	0.0356 (12)	0.0069 (10)	0.0064 (10)	0.0006 (11)
C12	0.0337 (11)	0.0262 (11)	0.0441 (13)	0.0062 (9)	0.0080 (10)	0.0096 (9)
C9	0.0358 (12)	0.0392 (12)	0.0330 (12)	0.0037 (9)	0.0025 (9)	0.0040 (9)
C4	0.0384 (12)	0.0417 (13)	0.0355 (12)	0.0007 (10)	0.0052 (10)	−0.0010 (10)
C5	0.0602 (16)	0.0482 (15)	0.0389 (14)	−0.0036 (12)	0.0011 (12)	−0.0052 (12)
C3	0.0425 (13)	0.0270 (11)	0.0426 (13)	0.0019 (9)	0.0033 (10)	−0.0024 (9)
C2	0.0370 (11)	0.0276 (11)	0.0368 (12)	0.0055 (9)	0.0034 (9)	0.0052 (9)
C21	0.0634 (17)	0.0437 (14)	0.0456 (15)	0.0030 (12)	0.0044 (13)	−0.0050 (12)
C20	0.0351 (12)	0.0410 (13)	0.0363 (12)	0.0014 (10)	0.0071 (10)	−0.0014 (10)
C6	0.0670 (18)	0.0696 (19)	0.0353 (14)	−0.0041 (15)	−0.0060 (13)	−0.0019 (13)
C14	0.0572 (15)	0.0338 (13)	0.0608 (16)	0.0148 (11)	0.0081 (13)	0.0226 (12)
C15	0.0487 (14)	0.0228 (11)	0.0625 (16)	0.0077 (10)	0.0170 (12)	0.0100 (11)
C26	0.0409 (13)	0.0531 (14)	0.0370 (13)	0.0148 (11)	0.0017 (10)	0.0127 (11)
C13	0.0532 (14)	0.0327 (12)	0.0490 (14)	0.0093 (10)	0.0009 (11)	0.0092 (11)
C17	0.0300 (10)	0.0259 (10)	0.0420 (12)	0.0033 (8)	0.0101 (9)	0.0069 (9)
C8	0.0578 (15)	0.0496 (15)	0.0413 (14)	0.0081 (12)	−0.0041 (12)	0.0095 (11)
C27	0.0367 (12)	0.0392 (12)	0.0399 (13)	0.0114 (9)	0.0024 (10)	0.0087 (10)
C24	0.0590 (17)	0.0692 (19)	0.0370 (14)	0.0096 (14)	−0.0001 (12)	0.0011 (13)
C7	0.0646 (17)	0.0703 (19)	0.0391 (14)	0.0113 (14)	−0.0051 (13)	0.0133 (13)
C23	0.086 (2)	0.073 (2)	0.0393 (15)	0.0053 (17)	−0.0039 (15)	−0.0150 (14)
N4	0.0364 (11)	0.0474 (12)	0.0519 (13)	0.0075 (9)	0.0045 (9)	0.0099 (10)
N5	0.0725 (18)	0.149 (3)	0.0665 (18)	0.0116 (18)	0.0128 (15)	0.0513 (19)

### Geometric parameters (Å, °)

**Table:**

Mn1—O2	1.8644 (15)	C25—C20	1.417 (3)
Mn1—O1	1.8799 (14)	C25—C26	1.425 (4)
Mn1—N1	1.9663 (17)	C12—C13	1.389 (3)
Mn1—N2	1.9672 (17)	C12—C17	1.405 (3)
Mn1—N3	2.2465 (19)	C9—C4	1.409 (3)
Mn1—O3	2.3905 (16)	C9—C8	1.411 (3)
N2—C18	1.309 (3)	C4—C5	1.416 (3)
N2—C17	1.419 (3)	C4—C3	1.418 (3)
O1—C1	1.309 (2)	C5—C6	1.361 (4)
N1—C11	1.306 (3)	C5—H5	0.9300
N1—C12	1.426 (3)	C3—C2	1.355 (3)
O2—C28	1.310 (3)	C3—H3	0.9300
C11—C10	1.419 (3)	C2—H2	0.9300
C11—H11	0.9300	C21—C20	1.405 (3)
O3—H3WA	0.824 (10)	C21—H21	0.9300
O3—H3WB	0.82 (3)	C6—C7	1.393 (4)
C1—C10	1.414 (3)	C6—H6	0.9300
C1—C2	1.420 (3)	C14—C15	1.380 (4)
C28—C19	1.409 (3)	C14—C13	1.381 (3)
C28—C27	1.425 (3)	C14—H14	0.9300
C19—C18	1.422 (3)	C15—H15	0.9300
C19—C20	1.458 (3)	C26—C27	1.341 (3)
C10—C9	1.458 (3)	C26—H26	0.9300
C16—C15	1.375 (3)	C13—H13	0.9300
C16—C17	1.388 (3)	C8—C7	1.367 (4)
C16—H16	0.9300	C8—H8	0.9300
C18—H18	0.9300	C27—H27	0.9300
N3—N4	1.180 (3)	C24—C23	1.359 (4)
C22—C21	1.369 (4)	C24—H24	0.9300
C22—C23	1.396 (4)	C7—H7	0.9300
C22—H22	0.9300	C23—H23	0.9300
C25—C24	1.413 (3)	N4—N5	1.163 (3)
			
O2—Mn1—O1	91.53 (6)	C17—C12—N1	115.35 (18)
O2—Mn1—N1	173.78 (7)	C4—C9—C8	117.2 (2)
O1—Mn1—N1	92.11 (7)	C4—C9—C10	119.0 (2)
O2—Mn1—N2	92.48 (7)	C8—C9—C10	123.8 (2)
O1—Mn1—N2	172.58 (6)	C9—C4—C5	120.2 (2)
N1—Mn1—N2	83.37 (7)	C9—C4—C3	119.6 (2)
O2—Mn1—N3	94.70 (7)	C5—C4—C3	120.2 (2)
O1—Mn1—N3	96.71 (7)	C6—C5—C4	121.1 (3)
N1—Mn1—N3	89.87 (7)	C6—C5—H5	119.5
N2—Mn1—N3	89.18 (7)	C4—C5—H5	119.5
O2—Mn1—O3	88.06 (6)	C2—C3—C4	121.7 (2)
O1—Mn1—O3	88.87 (6)	C2—C3—H3	119.2
N1—Mn1—O3	86.98 (6)	C4—C3—H3	119.2
N2—Mn1—O3	85.04 (6)	C3—C2—C1	120.8 (2)
N3—Mn1—O3	173.70 (6)	C3—C2—H2	119.6
C18—N2—C17	122.21 (18)	C1—C2—H2	119.6
C18—N2—Mn1	124.62 (15)	C22—C21—C20	121.4 (3)
C17—N2—Mn1	113.16 (13)	C22—C21—H21	119.3
C1—O1—Mn1	129.97 (13)	C20—C21—H21	119.3
C11—N1—C12	122.49 (18)	C21—C20—C25	117.3 (2)
C11—N1—Mn1	124.55 (15)	C21—C20—C19	124.1 (2)
C12—N1—Mn1	112.82 (14)	C25—C20—C19	118.7 (2)
C28—O2—Mn1	129.92 (14)	C5—C6—C7	118.8 (2)
N1—C11—C10	127.3 (2)	C5—C6—H6	120.6
N1—C11—H11	116.4	C7—C6—H6	120.6
C10—C11—H11	116.4	C15—C14—C13	120.7 (2)
Mn1—O3—H3WA	122 (3)	C15—C14—H14	119.7
Mn1—O3—H3WB	112 (3)	C13—C14—H14	119.7
H3WA—O3—H3WB	109 (2)	C16—C15—C14	120.3 (2)
O1—C1—C10	123.96 (18)	C16—C15—H15	119.8
O1—C1—C2	116.30 (18)	C14—C15—H15	119.8
C10—C1—C2	119.74 (18)	C27—C26—C25	122.1 (2)
O2—C28—C19	124.40 (19)	C27—C26—H26	119.0
O2—C28—C27	115.74 (19)	C25—C26—H26	119.0
C19—C28—C27	119.9 (2)	C14—C13—C12	119.7 (2)
C28—C19—C18	121.72 (19)	C14—C13—H13	120.2
C28—C19—C20	119.3 (2)	C12—C13—H13	120.2
C18—C19—C20	119.0 (2)	C16—C17—C12	119.6 (2)
C1—C10—C11	121.72 (19)	C16—C17—N2	125.5 (2)
C1—C10—C9	119.03 (19)	C12—C17—N2	114.96 (17)
C11—C10—C9	119.25 (19)	C7—C8—C9	121.1 (2)
C15—C16—C17	120.1 (2)	C7—C8—H8	119.4
C15—C16—H16	120.0	C9—C8—H8	119.4
C17—C16—H16	120.0	C26—C27—C28	120.8 (2)
N2—C18—C19	126.8 (2)	C26—C27—H27	119.6
N2—C18—H18	116.6	C28—C27—H27	119.6
C19—C18—H18	116.6	C23—C24—C25	121.1 (3)
N4—N3—Mn1	123.20 (16)	C23—C24—H24	119.5
C21—C22—C23	121.1 (3)	C25—C24—H24	119.5
C21—C22—H22	119.5	C8—C7—C6	121.6 (3)
C23—C22—H22	119.5	C8—C7—H7	119.2
C24—C25—C20	119.9 (2)	C6—C7—H7	119.2
C24—C25—C26	120.8 (2)	C24—C23—C22	119.2 (3)
C20—C25—C26	119.3 (2)	C24—C23—H23	120.4
C13—C12—C17	119.66 (19)	C22—C23—H23	120.4
C13—C12—N1	125.0 (2)	N5—N4—N3	179.3 (3)

### Hydrogen-bond geometry (Å, °)

**Table:**

*D*—H···*A*	*D*—H	H···*A*	*D*···*A*	*D*—H···*A*
O3—H3WA···N3^i^	0.82 (1)	2.10 (3)	2.911 (3)	167 (4)
O3—H3WB···O1^ii^	0.82 (3)	2.14 (3)	2.941 (2)	164 (4)
O3—H3WB···O2^ii^	0.82 (3)	2.57 (3)	3.130 (2)	127 (3)

Symmetry codes: (i) *x*−1, *y*, *z*; (ii) −*x*, −*y*, −*z*+2.
